# How Does Religiosity Influence Gambling? A Cross-Cultural Study Between Portuguese and English Youth

**DOI:** 10.1007/s10899-023-10269-0

**Published:** 2023-12-09

**Authors:** Filipa Calado, Mike Vernon, Filip Nuyens, Joana Alexandre, Mark D. Griffiths

**Affiliations:** 1 Department of Psychology, Nottingham Trent University, Nottingham, UK; 2 ISCTE - CIS/IUL - Lisbon University Institute, Lisbon, Portugal

**Keywords:** Youth gambling, Problem gambling, Religious affiliations, Religiosity, Cognitive distortions

## Abstract

Research has shown that religion can play a protective role in diverse risky behaviors among young people. However, very little is known about the effect of religion in gambling, especially among young problem gamblers. A strong moral belief regarding gambling may prevent adolescents and young adults engaging in gambling and developing problems. Nevertheless, some evidence suggests that religion might have an influence on gambling cognitive distortions (i.e., some religious beliefs might influence the conceptions of chance and luck, which may contribute to an increase in gambling participation). The present study examined the different effects that religion can have on gambling behavior, in two different cultural contexts (i.e., Portugal and England), characterized by different religious affiliations. A sample (n = 725) comprising Portuguese (n = 312) and English (n = 413) adolescents and young adults completed an online survey. The findings indicated that Portuguese youth were more religious than their English counterparts. Moreover, religiosity was associated with lower gambling engagement among participants in both samples. Mediation analyses also showed that the cognitive distortion of illusion of control mediated the relationship between religiosity and problem gambling among the Portuguese participants, and the interpretative bias was a significant mediator in the English sample. The study’s findings suggest that religion can have a protective role on gambling behaviors. However, further research is needed to explore the interactive role of religion and cognitive distortions.

## Introduction

Religion has always had an important role in human behavior (Van Tongeren, 2019). From a mental health perspective, religion can provide guidelines that help individuals to find a course or purpose in their lives (Koenig et al., 2012). Stresses and strains, as well as uncertainties of life, can be tolerated more easily by religious believers (Behere et al., 2013). In fact, many individuals turn to religion and spirituality when faced with stressful life events, and religious belief is associated with increased levels of posttraumatic growth (Sharma et al., 2017). A systematic review concluded that higher levels of religiosity are positively associated with indicators of psychological wellbeing (e.g., happiness, life satisfaction) and negatively associated with depression, suicide, and drug and alcohol abuse/dependence (Moreira-Almeida et al., 2006). Moreover, a growing body of research suggests that religion can have a protective effect on some health risky behaviors among adolescents and emerging adults. For instance, Fletcher and Kumar (2014) found that adolescents who reported religion as being important were less likely to use addictive psychoactive substances.

However, very little is known about the effect of religion in gambling, in particular youth problem gambling. Youth problem gambling is an emergent public health issue across many countries (Calado et al., 2017). Religious beliefs can have an impact on risky behavioral patterns that are developed during adolescence (e.g., gambling behavior). Uecker and Stokes (2016) found that adolescents who attended weekly religious services were less likely to have ever gambled. Moreover, in a study with 570 students from the American University of Beirut, Ghandour and El Sayed (2013) found that lower levels of practice of faith were associated with higher odds of lifetime gambling (both social and non-problematic gambling). Stronger associations between religion and gambling have been observed among Muslim students, whose faith does not permit gambling. Furthermore, Casey et al. (2011) conducted a study with 436 Canadian adolescents aged 13–16 years and the results showed that religiosity was a protective factor against involvement in gambling for both males and females.

These studies appear to show that religion can have an impact on the behavioral patterns that are formed during adolescence, such as gambling behavior. Young people’s religious and moral views are shaped by a number of factors, such as parents, school, community, and the society in which they live (Welte et al., 2017). A strong moral belief regarding risky behaviors such as gambling, may well be a protective factor and might prevent adolescents and young adults engaging in problematic gambling (Casey et al., 2011).

However, although religion and religious beliefs may prevent young people from engaging in problematic gambling behaviors, some evidence suggests that religion might have an influence on some gambling cognitive distortions, and may contribute to an increase in gambling participation (e.g., Browne et al., 2019; Williams et al., 2022). In fact, religion and gambling can be considered to have some common aspects, such as the belief that someone may have control over external and uncontrollable events (Binde, 2007). One of the most consistent predictors of gambling involvement and problematic gambling are gambling fallacies, in which individuals constantly believe they have the ability to control gambling outcomes (Toneatto, 1999). For instance, Moore and Ohtsuka (1997) showed that adolescents and young adults who gamble more often displayed more optimistic views about their chances of winning.

Moreover, some studies have found that religious beliefs and social values influence the conceptions of chance, faith, and probability (i.e., probabilistic thinking; Chassapis & Chatzivasileiou, 2008; Kim et al., 2018). Furthermore, adhering to some superstitious and religious beliefs, praying to win, performing rituals, and wearing religious medallions as lucky charms strengthen habits and can encourage the belief that an individual can increase their chances of winning (Toneatto, 1999). In a study conducted with Pacific Island mothers living in New Zealand, Bellringer et al. (2005) found that their involvement in traditional gifting to community and churches was associated with gambling behavior. In some of these communities, gambling for the church was considered acceptable. Similarly, a study conducted by Lam (2006) found a positive relationship between importance of faith and gambling frequency among individuals who played the lottery.

Some religious affiliations, such as Muslim and Protestant perceive gambling as a deviant behavior (Ellison & McFarland, 2011), whereas other affiliations, such as Roman Catholic Church have no sanctions against gambling (Binde, 2007). Moreover, individuals conceive differently the control of God and the causality of everyday events due to their religious views and cultural and social values (Chassapis & Chatzivasileiou, 2008). In fact, a study conducted by Amir and Williams (1999) with children aged 11–12 years in England (some of them of Asian origin), found that the culture and the social experiences of children influence their knowledge of probability, and a significant proportion of Muslim children had superstitions, and attributed outcomes of chance events to God.

Moreover, individuals from different cultural backgrounds can develop different gambling cognitions as a result of their unique upbringing. For example, Papineau (2005) reported that Chinese people perceive fate as something unavoidable and the outcome of a game is used to infer individuals’ destiny, including business prospects and love life. Therefore, culture-specific beliefs among Chinese gamblers might contribute and reinforce cognitive distortions such as the illusion of control. Therefore, it can be assumed that some cultures with specific religious affiliations characterized by stronger religious practices and through a greater importance of religion can hold different views about the causation of different phenomena, which can influence their involvement in gambling behavior. In other words, people who are religious may be more prone to the development of cognitive distortions.

Consequently, religious affiliation and religiosity might play important yet different roles in fostering and protecting against the development of problem gambling in different cultural groups. However, to the best of the present authors’ knowledge, very few studies have examined this differential effect of religion on the development of gambling and problematic gambling. Based on the aforementioned literature, there is a need for more rigorous methodologies and broader samples. Therefore, the present study tested a theoretical model to further knowledge concerning the different effects that religion can have on youth gambling behavior, in two different cultural contexts (i.e., Portugal and England). To the best of the present authors’ knowledge, no previous studies have simultaneously examined the role of religiosity in youth gambling behavior in samples from two different countries.

Although Portugal and England are two Western European countries, the two countries have profound variances in social structure, demographics, philosophies, cultural dimensions, and religion (Hofstede, 2001). In fact, Portugal is considered one of the most religious countries in Europe, with more than 84% of the population being members of the Roman Catholic Church (Instituto Nacional de Estatıstica, 2011; Teixeira, 2019). Compared with the Catholic Church, the numbers of all other religious groups remain relatively low. On the other hand, in England, according to the 2021 census, “no religion” was the second most common response, and less than half of the population described themselves as “Christian” (Office for National Statistics, 2022). However, according to the same data, those who identified as Christian were less likely than average to regularly attend a religious service or meeting. Therefore, the differences in religion in both countries may well influence the gambling behavior of their populations, and as Portuguese individuals seem to place more emphasis on the importance of religion (Teixeira et al., 2019) it was expected that the effect of religiosity on the gambling cognitions would be stronger in this context.

Therefore, the hypotheses for the present study were twofold. First, it was hypothesized that religiosity would have a direct and negative influence on gambling behavior, such that higher levels of religiosity would be associated with lower levels of problematic gambling behavior in both samples, English and Portuguese (H_1_). Second, it was hypothesized that higher levels of religiosity would have an indirect influence on gambling behavior, such that higher levels of religiosity would be associated with higher scores on the cognitive distortions (i.e., illusion of control, predictive control and interpretative bias, which according to research are the cognitive distortions more influenced by religion), which in turn would be associated with higher levels of problematic gambling among the Portuguese sample (H_2_).

## Method

### Participants, Procedure, and Ethics

The participants comprised 725 adolescents and young adults from Portugal and England, attending high schools and the first year of university. The Portuguese sample comprised 312 participants (mean age = 18.5 years; SD = 2.4), and the English sample comprised 413 participants (mean age = 19.1 years; SD = 1.8). Portuguese participants were recruited in Lisbon, whereas English participants were recruited in Nottingham. The institutional ethics committee of the research team’s university gave ethical approval for the study.

For each country, the sample comprised late adolescents and young adults. With regard to late adolescents, a contact was made between the first author and the headmaster of three schools, two private and one public in Lisbon (for the Portuguese sample), and a similar contact was made between the first author and two public schools in Nottingham (to recruit the English sample). Then, an information letter explaining the purpose of the study was sent to the school headmasters. If the headmaster provided permission, another letter was sent to students and their parents (if participants were minors). Only participants who provided their full informed consent participated in the study.

For recruiting young adults, some first-year college lecturers were contacted and after obtaining their permission for collecting data in their class, another letter was sent to their students. After participants provided their informed consent, they were allowed to participate in the study. Data were collected using a survey, completed on a voluntary basis in the classroom of the school or university. Besides the variables already mentioned, participants were also asked about other psychological variables, such as sensation seeking and attachment, which were analysed in another study (Calado et al., 2020). However, besides the data included in the surveys, participants were not asked for any other information.

### Measures

#### Sociodemographic Information and Gambling Frequency

Sociodemographic data were collected on age, gender, and religious affiliation. Participants were also asked to indicate how often they had gambled during the past year from 1 (“*never*”) to 6 (“*every day*”).

#### DSM-IV-Multiple Response-Juvenile (DSM-IV-MR-J)

The DSM-IV-MR-J is a psychometrically validated scale developed by Fisher (2000) for assessing youth problem gambling among those who have gambled during the past year. The scale contains nine items, and assesses a number of important variables related to youth problem gambling, such as progression and preoccupation, tolerance, withdrawal, and loss of control. The response categories comprise 1 = *“never”,* 2 = *“once or twice”,* 3 = *“sometimes”,* and 4 = *“often”.* Total score (range 0–9) was calculated by summing up the scores of all nine items. Participants who obtain a score of 0 or 1 are classified as social gamblers, a score of 2 or 3 indicates at-risk gambling, and a score of 4 or more indicates problem gambling. The present study used the validated Portuguese version of this scale (Calado et al., 2016) for the Portuguese sample. The Cronbach´s alpha of the scale was 0.72 in the English sample and 0.71 in the Portuguese sample.

#### Gambling Related Cognitions Scale (GRCS)

The 23-item Gambling Related Cognitions Scale (GRCS) developed by Raylu and Oei (2004) was used to assess gambling-related erroneous cognitions. This scale comprises five sub-scales, which correspond to different types of cognitive distortions: *gambling expectancies* (i.e., expected benefits from gambling); *illusion of control* (i.e., the perceived ability to control gambling outcomes); *predictive control* (i.e., the misattribution of cause-and-effect relationships to unlinked events); *inability to stop gambling* (i.e., the perceived inability to stop gambling behavior); and *interpretative bias* (i.e., an error of assessment, such as attributing wins to personal abilities). Higher scores on the GRCS indicate higher levels of cognitive distortions. For the present study, only the cognitive distortions of illusion of control, predictive control and interpretative bias were used as they were the only variables related to our research aims. In the present study, the instrument was translated and back translated to Portuguese to be administered to the Portuguese sample following standardized international guidelines (Beaton et al., 2000). For the Portuguese sample, the Cronbach alphas for the subscales interpretative bias, illusion of control, predictive control were respectively 0.86; 0.86 and 0.87. For the English sample, the Cronbach alphas for these sub-scales were respectively 0.80, 0.76 and 0.84.

#### Religiosity

Religiosity was assessed using a scale that was slightly adapted by the one developed by Johnson et al. (2001) and comprises four items. The first and third items assess the behavioral dimension of religiosity (e.g., frequency of attending religious services), on a 5-point Likert scale from 1 (*“never”*) to 5 (*“daily”*). The remaining two items assess the attitudinal dimension (e.g., importance of religion in someone’s life, often called religious salience), which were also measured on a 5-point Likert scale from 1 (*“Not important at all”*) to 5 (*“Very important”*). The instrument was translated and back translated to the Portuguese language following the guidelines outlined by Beaton (2000). The Cronbach’s alpha of the scale was 0.91 for the English sample and 0.92 for the Portuguese sample.

### Data Analysis

The data were analyzed descriptively in order to characterize each sample and to identify the religious affiliations and levels of religiosity in each country. Furthermore, to test the model in which religiosity will influence problematic gambling behavior both directly and indirectly through the cognitive distortions of interpretative bias, illusion of control and predictive control, mediation models were tested in lavaan (latent variable analysis) package, with maximum likelihood (ML) estimation. The analyses were conducted in SPSS and in R.

The models were estimated using the lavaan (latent variable analysis) package, with maximum likelihood (ML) estimation, using R (version 4.1.1) (R Core Team, 2018). After setting up the models using lavaan model syntax, the models were estimated using the sem() function before the summary() function is used for the examination of the results. The bootstrap method was conducted for testing the significance of the indirect effects (Bollen & Stine, 1990; Shrout & Bolger, 2002). The set.seed() function was used, specifying sample 123, to allow for the bootstrap simulations to simulate via the same sample. Six separate mediation models were estimated, in which religiosity was the predictor variable and problematic gambling behavior (PGB) was the outcome variable in all instances. The three mediators were examined separately across the mediation models, once for the English sample and once for the Portuguese sample.

Multiple model adjustment indicators [Comparative Fit Index (CFI), Tucker–Lewis Index (TLI), Root Mean Square Error of Approximation (RMSEA), and Standardised Root Mean Square Residual (SRMR)] were used to assess the goodness of fit of the proposed models in relation to the data. The CFI provides a measure of the relative improvement in fit when comparing the baseline model to the postulated model. It is typically considered that a CFI ≥ 0.95 indicates a good fit between the models (Hu & Bentler, 1999; West et al., 2012). The TLI (Tucker & Lewis, 1973) provides a measure of the amount of misfit per degree of freedom. Similar to the CFI, a TLI ≥ 0.95 indicates a good fit between the models (Hu & Bentler, 1999; West et al., 2012). In contrast, the RMSEA and SRMR indicates poor fit of the models, where lower values indicate a better fit. It is proposed that an RMSEA ≤ 0.1 is required for any reasonable consideration (Browne & Cudeck, 1992), with an RMSEA ≤ 0.06 considered more acceptable (Hu & Bentler, 1999). A SRMR ≤ 0.08 is considered an indication of a good fit (Hu & Bentler, 1999).

### Results

Descriptive statistics were conducted to understand the sociodemographic characteristics of each sample and religious affiliations in each country. The sociodemographic characteristics of each sample, as well as descriptive statistics of religious affiliations can be found respectively in Tables 1 and 2. As shown in Table 2, Roman Catholic was the predominant religious affiliation in the Portuguese, whereas in the English sample, the majority of individuals classed themselves as having no religion (or atheist). Moreover, the levels of religiosity were significantly higher in the Portuguese sample (*M* = 2.08, *SD* = 1.47) compared to the English sample (*M* = 1.47; *SD* = 0.76): *t*(516.56.) = 8.4, *p* < 0.001.

**Table 1:** Demographics background of participants

	Portuguese youth		English youth	
Gender	N	%	N	%
Male	142	45.5	150	36.3
Female	170	54.5	263	63.7
*Age*				
< 18 years	135	43.3	82	19.9
> = 18 years	177	56.7	331	80.1
*Qualification of parents*				
Primary school or less	29	9.3	0	0
Attendance of some secondary school	77	24.7	0	0
Completed secondary school	102	32.7	140	33.9
Some university	32	10.3	93	22.5
Completed university	71	22.8	180	43.6
*Gambling frequency*				
Most days	11	3.5	31	7.5
At least once per week	64	20.5	109	26.4
Once or twice a month	74	23.7	82	19.9
Less than once a month	163	52.2	191	46.2

**Table 2:** Religious affiliations in each country

England	%	Portugal	%
Church of England	17.1	Church of England	0
Roman catholic	7.2	Roman catholic	52.9
Islam	3.1	Islam	1.1
Judaism	0.7	Judaism	1.1
Hinduism	1.5	Hinduism	0
Buddhism	0.9	Buddhism	0
Other religion	5.1	Other religion	7.6
No religion	64.4	No religion	37.3

Exploratory correlation analysis was run separately between the Portuguese and English samples to examine any potential dissimilitude (Table 3). As can be seen, most correlations between the two samples were similar and showed the same degree of significance. Moreover, the association between illusion of control and religiosity was significant in the Portuguese sample but not in the English sample. This dissimilitude further supports the separate exploration of these two samples (Table 3).

**Table 3:** Correlations between the predictor, outcome, and mediator variables

Sample	Variable	Religiosity	IoC	PC	IB	PGB
Portugal	1. Religiosity	−				
	2. IoC	0.13*	−			
	3. PC	− 0.02	0.69***	−		
	4. IB	− 0.03	0.54***	0.68***	−	
	5. PGB	− 0.05	0.42***	0.58***	0.45***	−
England	1. Religiosity	−				
	2. IoC	0.01	−			
	3. PC	− 0.07	0.63***	−		
	4. IB	− 0.1	0.51***	0.63***	−	
	5. PGB	− 0.06	0.33***	0.46***	0.45***	−

*IoC* illusion of control, *PC* predictive control, *IB* interpretative bias, *PGB* problematic gambling behavior

*p < 0.05

***p < 0.001

**Fig. 1:**
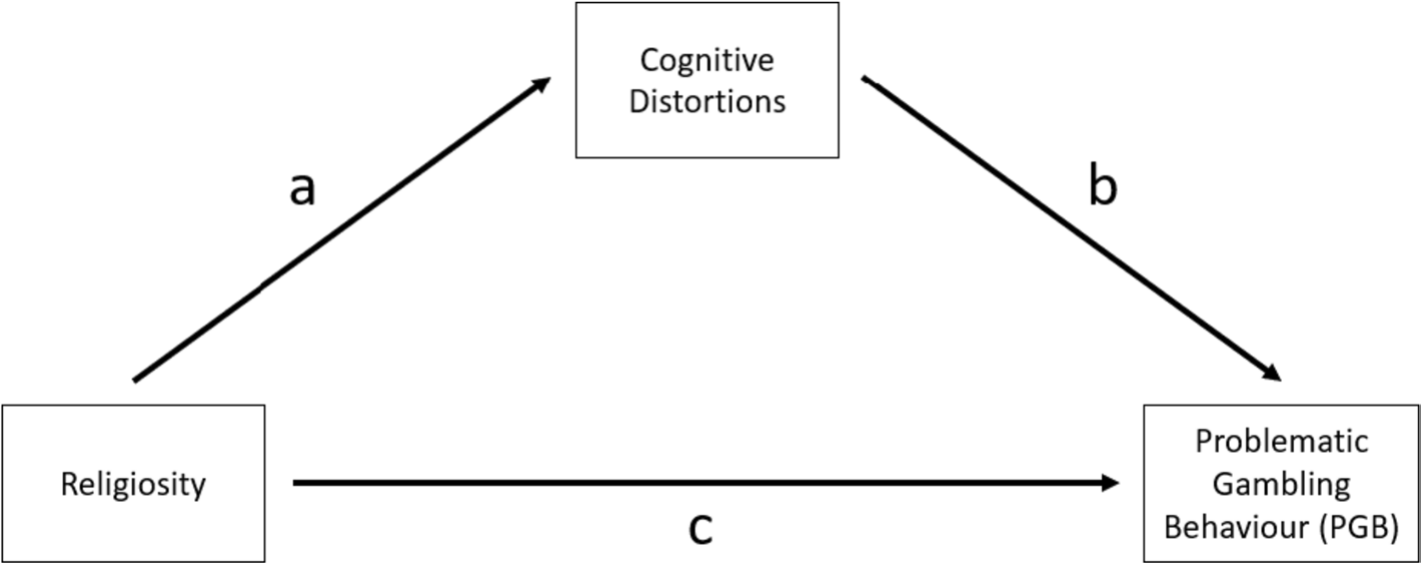
Mediation pathway for the relationship between Religiosity and problematic gambling behavior via the direct path (c) and the indirect path (a + b) of the moderator variables (cognitive distortions: illusion of control, predictive control or interpretative bias)

**Fig. 2:**
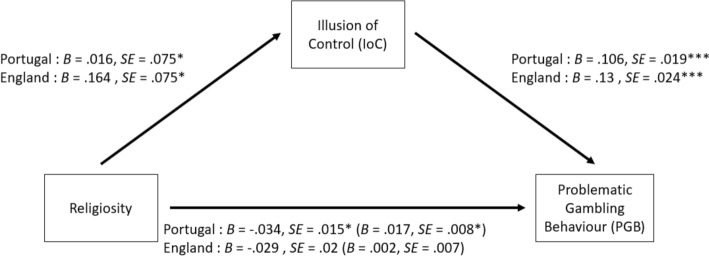
Parallel mediation model for the English and Portuguese samples featuring religiosity as the predictor variable, problematic gambling behavior as the outcome variable, and illusion of control as the mediator. The coefficients and standard errors for the indirect effect containing the mediator are shown in parentheses. *p < 0.05; **p < 0.01; ***p < 0.001

**Fig. 3:**
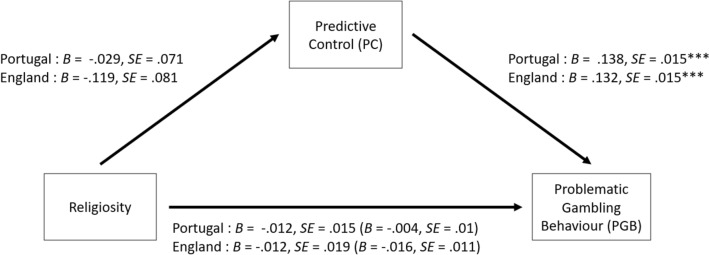
Parallel mediation model for the English and Portuguese samples featuring religiosity as the predictor variable, problematic gambling behavior as the outcome variable, and predictive control as the mediator. The coefficients and standard errors for the indirect effect containing the mediator are shown in parentheses. *p < 0.05; **p < 0.01; ***p < 0.001

**Fig. 4:**
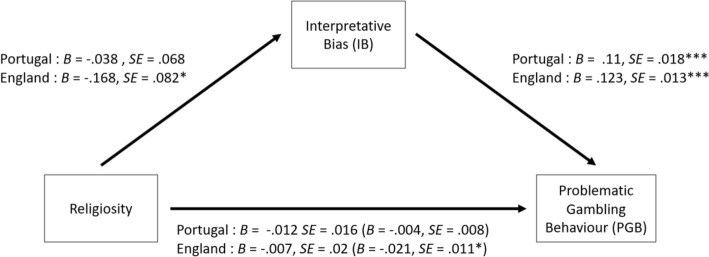
Parallel mediation model for the English and Portuguese samples featuring Religiosity as the predictor variable, problematic gambling behavior as the outcome variable, and Interpretative Bias as the mediator. The coefficients and standard errors for the indirect effect containing the mediator are shown in parentheses. *p < 0.05; **p < 0.01; ***p < 0.001

Subsequently, mediation analyses were conducted to test the mediating effect of each cognitive distortion in the relationship between religiosity and problematic gambling. More specifically, mediation models were estimated to examine if the predictor variable of religiosity (X) affected the outcome variable of problematic gambling behavior (Y) via a third variable (M), illusion of control, predictive control, or interpretative bias. The structure used for these models is illustrated in Fig. 1, in which there are two paths connecting the predictor and outcome variables. The direct path (path C) represents the predictor variable of religiosity affecting the outcome variable of problematic gambling behavior. The indirect path (path A + B) represents the predictor variable of religiosity affecting the mediator (i.e., illusion of control, predictive control, or interpretative bias), which in turn affects the outcome variable of problematic gambling behavior. Therefore, three mediation paths were conducted, which corresponded to the three cognitive distortions, which are illustrated in Figs. 2, 3 and 4.

#### Illusion of Control

In the Portuguese sample, religiosity significantly predicted problematic gambling behavior directly (β =  − 0.034, *SE* = 0.015,* Z* =  − 2.226, *p* = 0.026). This effect was significantly mediated by the Illusion of control whereby the indirect pathway of the relationship between religiosity and problematic gambling behavior via the Illusion of control was significant (β_[indirect]_ = 0.017, *SE* = 0.008, *Z* = 2.039, *p* = 0.041). The model adjustment indicators were adequate (χ^2^ = 70.844, *p* < 0.001, *CFI* = 1.00, *TLI* = 1.00, *RMSEA* < 0.01 (90% CI, *p* < 0.05), and *SRMR* < 0.01).

In the English sample, the direct effect of religiosity did not predict problematic gambling behavior (β =  − 0.029, *SE* = 0.02,* Z* =  − 1.468, *p* = 0.142). This effect was not significantly mediated by the illusion of control (β_[indirect]_ = 0.002, *SE* = 0.007, *Z* = 0.283, *p* = 0.777). The model adjustment indicators were adequate (χ^2^ = 49.16, *p* < 0.001, *CFI* = 1.00, *TLI* = 1.00, *RMSEA* < 0.01 (90%*CI*, *p* < 0.05), and *SRMR* < 0.01).

#### Predictive Control

Analysis of the Portuguese sample found that religiosity did not significantly predict problematic gambling behavior directly (β =  − 0.012, *SE* = 0.015,* Z* =  − 0.848, *p* = 0.396). There was a non-significant indirect effect, in which the relationship between religiosity and problematic gambling behavior was not significantly mediated via predictive control (β_[indirect]_ =  − 0.004, *SE* = 0.01,* Z* =  − 0.412, *p* = 0.681). The model adjustment indicators were adequate (χ^2^ = 130.59, *p* < 0.001, *CFI* = 1.00, *TLI* = 1.00, *RMSEA* < 0.01 (90% *CI*, *p* < 0.05), and *SRMR* < 0.01).

A similar pattern of results was found in the English sample. Religiosity did not significantly predict problematic gambling behavior directly (β =  − 0.012, *SE* = 0.019,* Z* = -0.621, *p* = 0.535), and the indirect effect was also non-significant (β_[indirect]_ = -0.016, *SE* = 0.0011,* Z* = -1.493, *p* = 0.15). The model adjustment indicators were adequate (χ^2^ = 101.447, *p* < 0.001, *CFI* = 1.00, *TLI* = 1.00, *RMSEA* < 0.01 (90%*CI*, *p* < 0.05), and *SRMR* < 0.01).

#### Interpretative Bias

A non-significant direct effect was found for religiosity predicting problematic gambling behavior in the Portuguese sample (β =  − 0.012, *SE* = 0.016,* Z* =  − 0.763, *p* = 0.446), with a non-significant indirect effect also found (β =  − 0.004, *SE* = 0.008,* Z* =  − 0.548, *p* = 0.584). Interpretative bias did not significantly mediate the relationship between religiosity and problematic gambling behavior. The model adjustment indicators were adequate (χ^2^ = 70.003, *p* < 0.001, *CFI* = 1.00, *TLI* = 1.00, *RMSEA* < 0.01 (90%*CI*, *p* < 0.05), and *SRMR* < 0.01).

In the English sample, the direct effect of religiosity did not significantly predict problematic gambling behavior (β =  − 0.007, *SE* = 0.02,* Z* =  − 0.33, *p* = *0*.742). This effect was significantly mediated by interpretative bias (β*(indirect)* =  − 0.021, *SE* = 0.011,* Z* =  − 1.962, *p* = 0.05). The model adjustment indicators were adequate (χ^2^ = 98.845, *p* < 0.001, *CFI* = 1.00, *TLI* = 1.00, *RMSEA* < 0.01 (90%*CI*, *p* < 0.05), and *SRMR* < 0.01).

## Discussion

To the best of the present authors’ knowledge, the present study is the first to examine the differential effect of religiosity on problematic gambling behavior among adolescents and young adults, as well as the indirect effect through cognitive distortions in two different samples. First of all, it can be observed some differences between the two samples: the English sample is composed by older participants, and parents have higher levels of qualifications in comparison to the Portuguese sample. Moreover, the religious affiliation most predominant in the Portuguese sample was Roman Catholic, whereas in the English sample most participants classed themselves as non-religious.

Regarding the direct effect of religiosity, the findings of the present study showed that for both English and Portuguese samples there was a negative association between religiosity and problematic gambling, although this association did not always reach the statistical significance. In fact, this association only assumed statistical significance for the Portuguese sample. However, with regards the mediating effect of cognitive distortions, the cognitive distortion of illusion of control was a significant mediator in the Portuguese sample. Moreover, the interpretative bias was a significant mediator in the relationship between religiosity and problematic gambling in the English sample.

The findings related to the direct effect of religiosity on problematic gambling are in line with previous research on the field, who also showed a negative association between religiosity and problematic gambling. For example, Fletcher and Kumar (2014) found that religiosity can have a protective effect on some health risky behaviors, such as substance abuse among young people. In addition, other studies (e.g., Casey et al., 2011; Uecker & Stokes, 2016) specifically found that attending religious services and following some religious traditions can reduce the likelihood of gambling involvement. These findings can be explained by the fact that many religious institutions in Europe and around the world frame gambling and other risky behaviors, such as drinking alcohol, taking drugs and using pornography as sinful (Beyerlein & Sallaz, 2017). Many scholars have argued that religiosity can help to shape moral values regarding risky behaviors, which might prevent young people from engaging in potentially problematic behaviors, such as gambling (Casey et al., 2011). The finding that the negative association between religiosity and problematic gambling assumed the statistical significance in the Portuguese sample can be explained by the fact that the levels of religiosity are significantly higher in this sample.

Moreover, when examining the mediating effect of cognitive distortions, only the illusion of control significantly mediated the relationship between religiosity and problematic gambling in the Portuguese sample. Analyzing the data, it was found that religiosity showed a positive significant association with the illusion of control, which in turn influenced positively problematic gambling behavior. This finding is in line with previous research that supports that religiosity may play a positive influence in the development of cognitive distortions (e.g., Browne et al., 2019; Chassapis & Chatzivasileiou, 2008). This finding may also be explained by the fact that the Portuguese sample showed higher levels of religiosity in comparison with the English one. In fact, it is important to note that the illusion of control concerns the belief that if an individual engaged in specific rituals and behaviors, this will increase their chances of winning.

According to Toneatto (1999), in societies with higher levels of religiosity, adhering to some religious beliefs, and performing some rituals can encourage the belief that an individual can increase their chances of winning, and that if such individuals behave and go to the church, God *“will enable [them] to win*”. Moreover, individuals who think that life events can be influenced by a higher power will also be more likely to hold the belief that the same higher power can interfere in the outcome of gambling games (Kim et al., 2018). In addition, according to some scholars (e.g., Binde, 2007), the Roman Catholic Church, which was the most predominant religious affiliation in the Portuguese sample, does not label gambling as a dangerous sin in comparison with a Protestant society because many revenues of gambling games go to the Catholic church, which also helps to explain why the illusion of control, which is a cognitive distortion that can be more associated with this religious affiliation, appears to influence problematic gambling.

Another interesting finding was the significant mediating effect of the interpretative bias in the relationship between religiosity and problematic gambling in the English sample. Analyzing this effect, it can be noted that religiosity is significantly negatively associated with interpretative bias, which in turn will significantly influence problematic gambling behaviour. Examining this cognitive distortion in further detail, it can be observed that it concerns the beliefs about attributing wins to personal abilities and skills and remembering how much money was won in the past in order to continue to engage in gambling behaviour. These values of winning money are less in line with religious moral values and higher levels of religiosity. As the English sample showed lower levels of religiosity, this might explain the significant mediating effect of this cognitive distortion. However, these associations need to be further explored in future research.

The study’s findings showed no significant mediating effect of the predictive control in the relationship between religiosity and problematic gambling. Although these findings need to be further examined in future research, the results seem to suggest that not all cognitive distortions explain the relationship between religiosity and gambling involvement. In fact, predictive control is related to the misattribution of cause-and-effect relationships to unlinked events, such as that losses will be followed by a series of wins. Therefore, it seems that these cognitive distortions, due to their nature, are not influenced by the levels of religiosity. The findings indicated that only the cognitive distortions of illusion of control and interpretative bias might play a major role in the conceptions of chance and luck, which can influence someone’s decisions to gamble.

The findings of the present study also contain some theorical and practical implications. Future research on gambling should incorporate religious affiliations and religiosity in their models as both a risk and protective factor for predicting youth problematic gambling. Moreover, as the present study suggests that religiosity may protect young people from engaging in problematic gambling, in specific cultures and communities, attendance of religious services could be encouraged. However, as religiosity may also influence the development of specific cognitive distortions, more religious individuals should also be advised that importance of faith and commitment to God will not affect the outcomes of a gambling game.

### Strengths and Limitations

Although the present study has some strengths, such as the novelty of testing a model for predicting youth problem gambling in two different cultural contexts, and the examination of previously unexplored relationships between religiosity, cognitive distortions and problematic gambling, it is not without limitations. These should be taken into account when interpreting the findings. For instance, the present study exclusively utilized self-report data, which are prone to well-known biases, such as social desirability. In addition, the cross-sectional nature of the study does not allow the determination of cause-and-effect relationships. Future research should address this limitation by using longitudinal designs, which would capture measures of religiosity and gambling involvement over time. Moreover, the present study also comprised a convenience sample, and therefore it is not representative of youth populations in either country. Furthermore, religion is a multidimensional concept (Mutti-Packer et al., 2017), which involves two dimensions, a behavioral and attitudinal component, which were not explored separately in the present study. In fact, attending religious services can be considered a behavioral or community aspect of religiousness, whereas the extent to which someone internalizes their faith and shows the importance of religion in their life concerns the attitudinal aspect of religion. Therefore, further research should examine these dimensions to better understand which aspects of religion play a more robust influence in protecting young people from gambling involvement.

### Conclusion

The findings of the present study showed that the influence of religiosity on problematic gambling is complex. Overall, the results indicated that religiosity appears to protect young people from engaging in problematic gambling behaviors, which confirms previous research. Moreover, the findings appear to indicate that other aspects of religiosity might influence the development of cognitive distortions because it encourages the acquisition of transcendental beliefs that might increase perceived control and risk-taking. Therefore, there is the need to conduct additional research to further knowledge on the complex relationships between gambling and religiosity. The present study is novel in providing insight into the effect on religiosity on gambling behavior and generates important avenues for future research.

## Data Availability

The data are available from the first author upon reasonable request.
